# The Pattern of Bacterial Infections Among Chronic Suppurative Otitis Media Cases at a Tertiary Care Centre in North-East India

**DOI:** 10.7759/cureus.60371

**Published:** 2024-05-15

**Authors:** Shankhadhwaj Borah, Rupam Borgohain, Rupanjita Sangma, Narayan C Sharma, Putul Mahanta, Sudarshana B Khanikor, Jilimili Devi

**Affiliations:** 1 Otolaryngology, Sonari District Hospital, Sonari, IND; 2 Otolaryngology, Tezpur Medical College and Hospital, Tezpur, IND; 3 Otolaryngology, Assam Medical College and Hospital, Dibrugarh, IND; 4 Pediatrics, PA Sangma International Medical College and Hospital, Khanapara, IND; 5 Forensic Medicine and Toxicology, Nalbari Medical College and Hospital, Nalbari, IND; 6 Pharmaceutical Sciences, University of Science and Technology, Meghalaya, Baridua, IND; 7 Biochemistry, Jorhat Medical College and Hospital, Jorhat, IND

**Keywords:** spectrum of infecting organisms, otorrhea, drug sensitivity, bacterial infection, chronic otitis media

## Abstract

Background and objectives: Chronic suppurative otitis media (CSOM) is a chronic inflammation of the mucoperiosteal lining of the middle ear cleft, presenting with recurrent ear discharge through a tympanic membrane perforation. The present study aims to assess the spectrum of bacterial infection among CSOM cases and detect the isolated organism’s antibiotic sensitivity pattern.

Methods: The prospective hospital-based observational study was conducted from June 2021 to June 2022 and included 94 CSOM cases. An aural swab of the ear discharge was collected from each patient under aseptic precautions. The swab was utilized for Gram’s staining and the aerobic bacterial pathogen culture. The organisms isolated were tested for antibiotic sensitivity using the Kirby-Bauer disc diffusion method.

Results: The most affected age group was the second decade of life (27.7%, n=26), with a male-to-female ratio of 1.35:1. The mean duration of ear discharge was 24.0±14.7 months, mostly mucoid ear discharge (39.4%, n=37). Among gram-positive bacteria, methicillin-resistant *Staphylococcus aureus* was isolated in 16 (17.0%) cases. *Pseudomonas aeruginosa* was the most isolated gram-negative bacteria strain in 26 (27.7%) cases. Cotrimoxazole (67.7%, n=21) had the highest sensitivity towards gram-positive bacteria isolates. Amongst gram-negative bacteria, amikacin and ciprofloxacin were the most sensitive, with 78.0% (n=39) susceptibility.

Conclusion: Evaluating the spectrum of infecting organisms of CSOM and their antibiotic sensitivity may help initiate prompt treatment with the appropriate antibiotic regimen, thereby preventing future complications.

## Introduction

Chronic suppurative otitis media (CSOM) is one of the frequent causes of ear infection in underdeveloped countries, notably in Southeast Asian countries, and is defined by chronic inflammation of the mucoperiosteal lining of the middle ear cleft and mastoid cavity. It presents with recurrent ear discharge or otorrhoea through a tympanic membrane perforation. CSOM is characterized by permanent anomalies of the pars tensa or flaccida, most likely caused by preceding acute otitis media, negative middle ear pressure, or otitis media with effusion. The condition commonly begins early in life as a spontaneous tympanic membrane perforation caused by an acute middle ear infection or as a complication of secretory otitis media.

The complex multifactorial disorders have no specific genetic flaw, making it challenging to identify the genes responsible for the condition [1]. It is also triggered by numerous episodes of acute otitis media, persistent middle ear infection, allergies or other chronic inflammatory stimuli [2]. The prevalence of the condition is higher in underdeveloped and poverty-stricken countries. People in low-hygienic and overpopulated conditions are at the highest risk [3]. Eustachian tube incompetency is a leading cause of middle ear diseases among children, including otitis media [4]. Various factors are involved in developing CSOM, including genetic, infectious and environmental factors [5]. If left untreated, it may lead to life-threatening complications, including septicaemia, meningitis, brain abscess and facial palsy [6].

Complications and fatality rates have significantly reduced recently due to the availability of proper antibiotics, improved diagnostics and multidisciplinary treatment approach. However, managing CSOM is debated and prone to variations, especially in underdeveloped countries, due to the wide diversity in the prevalence and antibiogram of the causative organisms [7]. Microbial culture and sensitivity aid in deciding the appropriate treatment regimen and minimization of resistant bacterial strains [8]. The objectives of the present study were to assess the spectrum of bacterial infection and to detect the drug sensitivity pattern of the isolated organisms among CSOM cases.

## Materials and methods

This prospective hospital-based observational study, conducted in the Department of Otorhinolaryngology, Assam Medical College and Hospital (AMCH), Dibrugarh, from June 2021 to June 2022, was guided by stringent ethical considerations. Ethical clearance was obtained from the Institutional Ethics Committee of AMCH vide ref. no.: AMC/EC/PG/5525. The cases were selected irrespective of age, sex, caste, religion, duration of illness and severity of conditions. Prior informed consent was obtained from the patients, ensuring their autonomy and respect for their rights.

Inclusion and exclusion criteria

Patients of CSOM with tympanic membrane perforation giving consent for the study, who were willing to manage both conservative and operative ways, follow the medical advice and come at regular intervals for follow-up were included.

Cases with otitis externa, impacted wax, and foreign body ear were excluded. Those with anaerobic bacterial pathogens were also excluded. Patients refusing to give consent for the study were also excluded.

All cases underwent a comprehensive and meticulous history taking, followed by a thorough general physical examination and an in-depth examination of the ear, nose, and throat. The relevant details were meticulously recorded. Specific investigations included audiological tests, pure tone audiometry (PTA), examination under a microscope, aural swab for culture sensitivity and radiological imaging (high-resolution CT (HRCT) temporal-mastoid).

Aural ear discharge swabs were meticulously taken from each patient using aseptic techniques to ensure no surface contamination. Only cases with no systemic or local treatment in the form of ear drops for the last seven days were selected. A swab was used for Gram staining and inoculated on nutrient agar, blood agar, and MacConkey agar to culture the aerobic bacterial pathogen. The isolated organisms were tested for antibiotic sensitivity on Mueller-Hinton agar using the Kirby-Bauer disc diffusion method according to Clinical and Laboratory Standards Institute (CLSI) antimicrobial susceptibility testing standards.

Statistical analysis

The obtained data was tabulated in a Microsoft Excel Worksheet (2010; Microsoft® Corp., Redmond, WA, USA), and the analysis was done with the Statistical Package for the Social Sciences (IBM SPSS Statistics for Windows, IBM Corp., Version 20.0, Armonk, NY). The data were represented as tables and graphs using descriptive statistical methods. The categorical variables were defined using frequencies and percentages. Continuous data were represented as mean± standard deviation.

## Results

The study included 94 cases of CSOM. The mean age of involvement was 30.5±16.3 years. The most commonly affected age group was the second and third decades of life, constituting 50 (53.2%) cases. The male-to-female ratio was 1.35:1. An incidental matching of data with 50% (n=47) involvement of either the right or left ear and none of the cases with bilateral involvement was observed among the patients, as shown in Table 1.

**Table 1:** Characteristics of patients The data has been represented as the frequency of patients (n) and percentage (%).

Characteristics	Categories	Frequency (n=94)
Age group (in years)	0-10	6 (6.4%)
	11-20	26 (27.7%)
	21-30	24 (25.5%)
	31-40	17 (18.1%)
	41-50	8 (8.5%)
	51-60	7 (7.4%)
	>60	6 (6.4%)
Gender	Male	54 (57.5%)
	Female	40 (42.5%)
The side of the ear involved in the presentation	Right	47 (50.0%)
	Left	47 (50.0%)
	Bilateral	0 (0.0%)

**Figure 1:**
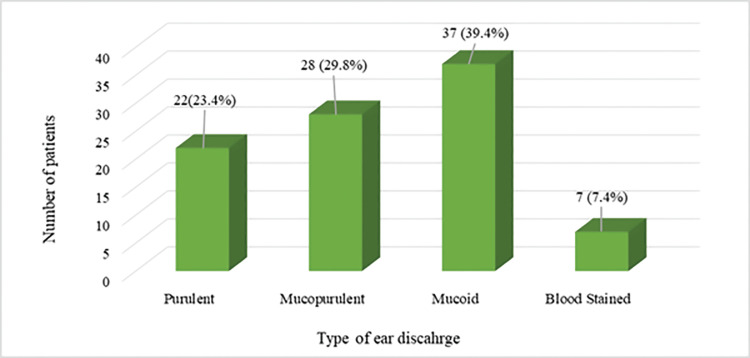
Type of ear discharge among the cases The data has been represented as the frequency of patients (n) and percentage (%).

In most cases, mucoid ear discharge was prevalent in 37 (39.4%) cases, followed by mucopurulent ear discharge in 28 (29.8%) cases. A minimum of seven (7.4%) cases had blood-stained ear discharge, as shown in Figure 1.

**Figure 2:**
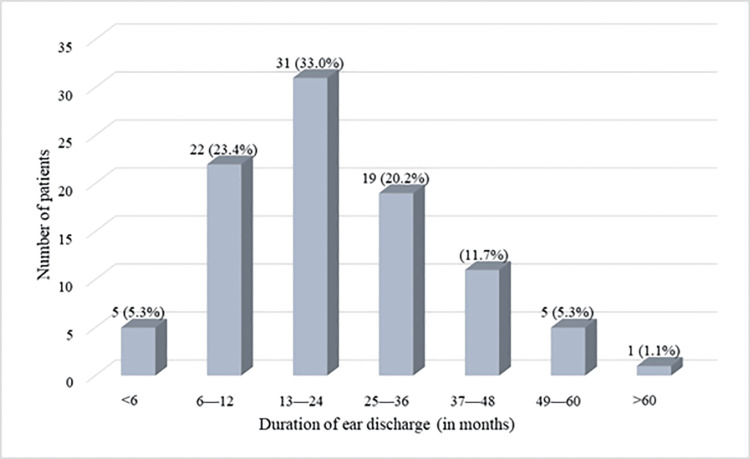
Duration of ear discharge among the cases The data has been represented as the frequency of patients (n) and percentage (%).

In most cases (n=31, 33.0%), ear discharge was prevalent for 13-24 months with a mean duration of 24.0±14.7 months. Only one (1.1%) of the cases had otorrhea for over 60 months, while the discharge duration was less than six months in five (5.3%) cases (Figure 2).

Of the 94 cases, 45 (47. 9%) had air conduction more than bone conduction, and 49 (52.1%) had bone conduction more than air conduction in the right ear. Similarly, 49 cases (52.13%) in the left ear had air conduction more than bone conduction, and 45 cases (47.87%) had bone conduction more than air conduction. Weber lateralized to the left side was found in 47 cases (50%). In comparison, 47 cases (50%) had Weber lateralized to the right ear, with clinical manifestations of otorrhoea and conductive hearing loss, as shown in Table 2.

**Table 2:** Tuning fork test findings of the 94 cases The data has been represented as the frequency of patients (n) and percentage (%).

Tuning Fork Test	Frequency (%)	Frequency (%)
Rinne Test	Right ear	Left ear
AC>BC	45 (47.9%)	49 (52.1%)
BC>AC	49 (52.1%)	45 (47.9%)
Weber’s test	Lateralized to Left	Lateralized to Right
	47 (50.0%)	47 (50.0%)

Medium central perforation was found in a total of 38 cases (40.4%), while sub-total tympanic membrane perforation was found in 34 cases (36.2%) and large central perforation consisted of 23.4% of cases respectively (Table 3).

**Table 3:** Findings of otoscopy or examination under microscope (EUM) of the 94 cases The data has been represented as the frequency of patients (n) and percentage (%). EUM: examination under microscope

Findings of Otoscopy or EUM	Right Ear (%) N= 47	Left Ear (%) N=47	Total (%) N=94
Medium Central Perforation	18 (19.1%)	20 (21.3%)	38 (40.4%)
Large Central Perforation	10 (10.6%)	12 (12.8%)	22 (23.4%)
Sub-Total Perforation	19 (20.2%)	15 (16.0%)	34 (36.2%)

The majority of 46 cases (48.9%) had air conduction in the 31-40 dB range, followed by 29 (30.8%) cases in the 41-50 dB range. At the same time, the majority of 49 cases (52.13%) had bone conduction at 11-20 dB. The mean± standard deviation of the air-bone gap was 26.6±6.7 dB (Table 4).

**Table 4:** Pure tone audiometry (PTA) findings The data has been represented as the frequency of patients (n) and percentage (%).

Pure Tone Audiometry (dB)	Air Conduction	Bone Conduction	Air Bone Gap
	Frequency (%)	Frequency (%)	Frequency (%)
0-10	0 (0.0%)	42 (44.7%)	0 (0.0%)
11-20	0 (0.0%)	49 (52.1%)	20 (21.3%)
21-30	16 (17.0%)	3 (3.2%)	47 (50.0%)
31-40	46 (48.9%)	0 (0.0%)	26 (27.7%)
41-50	29 (30.8%)	0 (0.0%)	1 (1.0%)
51-60	3 (3.2%)	0 (0.0%)	0 (0.0%)
Total	94 (100.0%)	94 (100.0%)	94 (100.0%)

As shown in Table 5, the bacterial infection pattern in CSOM cases showed a varying spectrum of isolated organisms. The Gram staining revealed a predominance of gram-negative bacteria, accounting for 50 cases (53.2%), followed by gram-positive bacteria in 31 cases (33.0%). Of the 94 cases, 11 (11.7%) were sterile and yeast-like fungi were isolated in two cases. Out of the 31 cases with gram-positive bacteria strains, *Staphylococcus aureus* was found in 30 cases, of which 16 were methicillin-resistant *S. aureus* (17.0%). *Streptococcus pyogenes* was isolated in one case (1.1%). Among the gram-negative bacteria strains, the most common isolated organism was *Pseudomonas aeruginosa* (n=26; 27.7%), followed by *Proteus mirabilis* (n=9; 9.6%), *Klebsiella pneumoniae* (n=5; 5.3%) and *Acinetobacter baumannii *(n=4; 4.3%). Other gram-negative bacterial strains isolated were *Citrobacter freundii*, *Escherichia coli*, *Klebsiella oxytoca* and *Achromobacter xylosoxidans* in a few numbers of cases.

**Table 5:** Distribution of isolated organisms The data has been represented as the frequency of patients (n) and percentage (%).

Type of Organisms	Name of Organisms	Frequency (%)
Gram-positive bacteria	Non-methicillin-resistant *Staphylococcus aureus*	14 (14.9%)
	Methicillin-resistant *S. aureus*	16 (17.0%)
	Streptococcus pyogenes	1 (1.1%)
	Total	31 (33.0%)
Gram-negative bacteria	Pseudomonas aeruginosa	26 (27.7%)
	Klebsiella pneumoniae	5 (5.3%)
	Proteus mirabilis	9 (9.6%)
	Acinetobacter baumannii	4 (4.3%)
	Escherichia coli	2 (2.1%)
	Achromobacter xylosoxidans	1 (1.1%)
	K. oxytoca	1 (1.1%)
	Citrobacter freundii	2 (2.1%)
	Total	50 (53.2%)
Sterile		11 (11.7%)
Yeast (Fungus)		2 (2.1%)
Total		94 (100.0%)

Cotrimoxazole (67.7%, n=21) was found to be the most sensitive antibiotic in gram-positive bacteria, trailed by clindamycin (48.4%, n=15), ciprofloxacin (45.2%, n=14) and azithromycin (41.9%, n=13). Linezolid (35.5%, n=11), gentamycin (35.5%, n=11) and tetracycline (38.7%, n=12) also showed fair sensitivity towards the gram-positive specimens. On the other hand, amikacin (58.1%, n=18), amoxicillin-clavulanic acid (51.6%, n=16), mupirocin (51.6%, n=16) and tobramycin (45.2%, n=14) showed high resistance towards gram-positive bacteria isolates. Imipenem, doxycycline and piperacillin-tazobactam showed no sensitivity towards gram-positive bacteria, but imipenem (77.4%, n=24) and piperacillin-tazobactam (83.9%, n=26) showed the highest intermediate sensitivity. Levofloxacin (58.1%, n=18) and meropenem (58.1%, n=18) were also intermediate sensitive towards these organisms.

Amongst gram-negative bacteria, amikacin and ciprofloxacin were the most sensitive, with 78.0% susceptibility (n=39), followed by levofloxacin (32.0%, n=16), amoxicillin-clavulanic acid (28.0%, n=14), cotrimoxazole (26.0%, n=13), piperacillin-tazobactam (24.0%, n=12) and gentamycin (24.0%, n=12). Imipenem was sensitive in 22% of cases (n=11), while tobramycin was sensitive in 18% of cases (n=9). Doxycycline (12%, n=6), linezolid (10%, n=5) and meropenem (6%, n=3) were found to be sensitive in a few numbers of cases. Mupirocin (88%, n=44) and azithromycin (60%, n=30) showed highest resistance towards the gram-negative bacteria strains. Tetracycline was sensitive in 4% of cases (n=2), while intermediate sensitivity in 54% (n=27). Clindamycin didn’t show sensitivity to any organism. It was found to be intermediate in 24 (48%) cases and resistant in 26 (52%) cases (Table 6).

**Table 6:** Culture and antimicrobial sensitivity pattern of bacteria isolated from chronic suppurative media patients The data has been represented as the frequency of patients (n) and percentage (%).

Antibiotic	Gram-Positive Bacteria (n=31)			Gram-Negative Bacteria (n=50)		
	Resistant	Susceptible	Intermediate	Resistant	Susceptible	Intermediate
Amikacin	18 (58.1%)	1 (3.2%)	12 (38.7%)	7 (14.0%)	39 (78.0%)	4 (8.0%)
Amoxicillin+clavulanic acid	16 (51.6%)	2 (6.4%)	13 (41.9%)	23 (46.0%)	14 (28.0%)	13 (26.0%)
Azithromycin	12 (38.7%)	13 (41.9%)	6 (19.3%)	30 (60.0%)	2 (4.0%)	18 (36.0%)
Ciprofloxacin	7 (22.6%)	14 (45.2%)	10 (32.2%)	4 (8.0%)	39 (78.0%)	6 (12.0%)
Clindamycin	8 (25.8%)	15 (48.4%)	8 (25.8%)	26 (52.0%)	0 (0.0%)	24 (48.0%)
Cotrimoxazole	8 (25.8%)	21 (67.7%)	2 (6.4%)	28 (56.0%)	13 (26.0%)	9 (18.0%)
Doxycycline	9 (29.0%)	0 (0.0%)	22 (71.0%)	19 (38.0%)	6 (12.0%)	25 (50.0%)
Gentamycin	7 (22.6%)	11 (35.5%)	13 (41.9%)	12 (24.0%)	12 (24.0%)	26 (52.0%)
Imipenem	7 (22.6%)	0 (0.0%)	24 (77.4%)	13 (26.0%)	11 (22.0%)	26 (52.0%)
Levofloxacin	12 (38.7%)	1 (3.2%)	18 (58.1%)	10 (20.0%)	16 (32.0%)	24 (48.0%)
Linezolid	7 (22.6%)	11 (35.5%)	13 (41.9%)	11 (22.0%)	5 (10.0%)	34 (68.0%)
Meropenem	12 (38.7%)	1 (3.2%)	18 (58.1%)	24 (48.0%)	3 (6.0%)	23 (46.0%)
Mupirocin	16 (51.6%)	10 (32.2%)	5 (16.1%)	44 (88.0%)	1 (2.0%)	5 (10.0%)
Piperacillin-tazobactum	5 (16.1%)	0 (0.0%)	26 (83.9%)	9 (18.0%)	12 (24.0%)	29 (58.0%)
Tetracycline	8 (25.8%)	12 (38.7%)	11 (35.5%)	21 (42.0%)	2 (4.0%)	27 (54.0%)
Tobramycin	14 (45.2%)	2 (6.4%)	15 (48.4%)	19 (38.0%)	9 (18.0%)	21 (42.0%)

**Figure 3:**
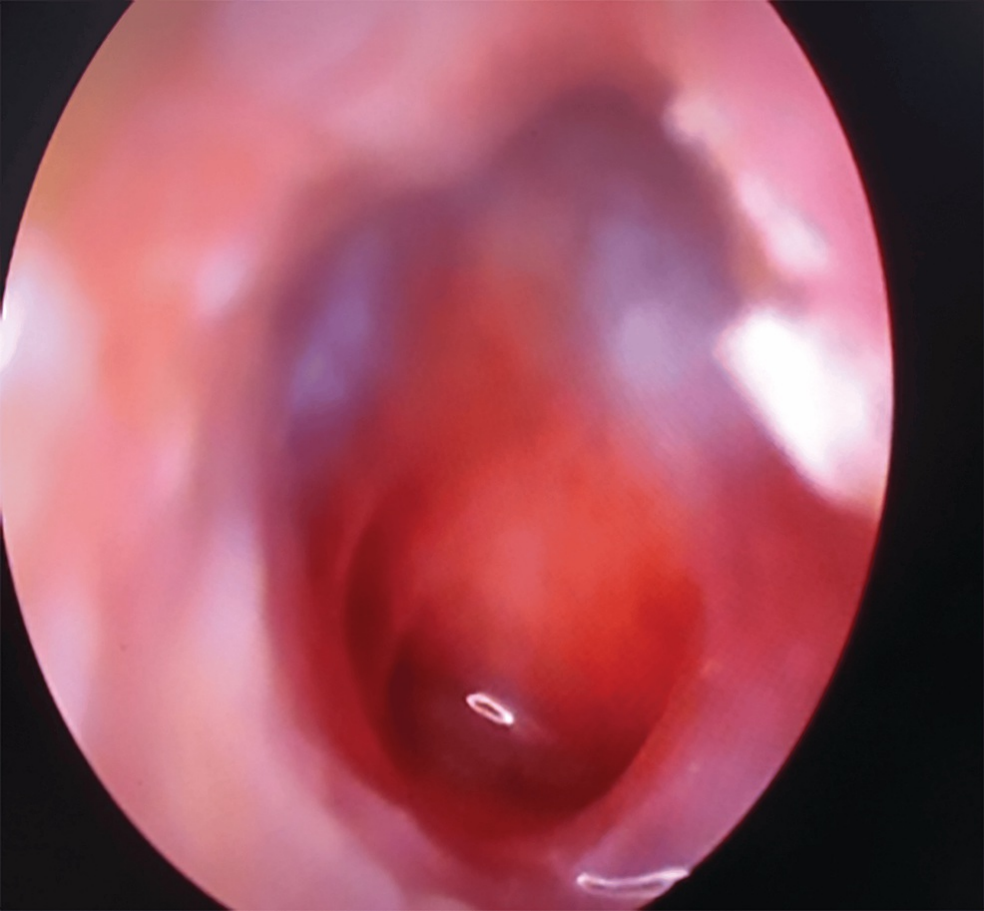
Large central tympanic membrane perforation

**Figure 4:**
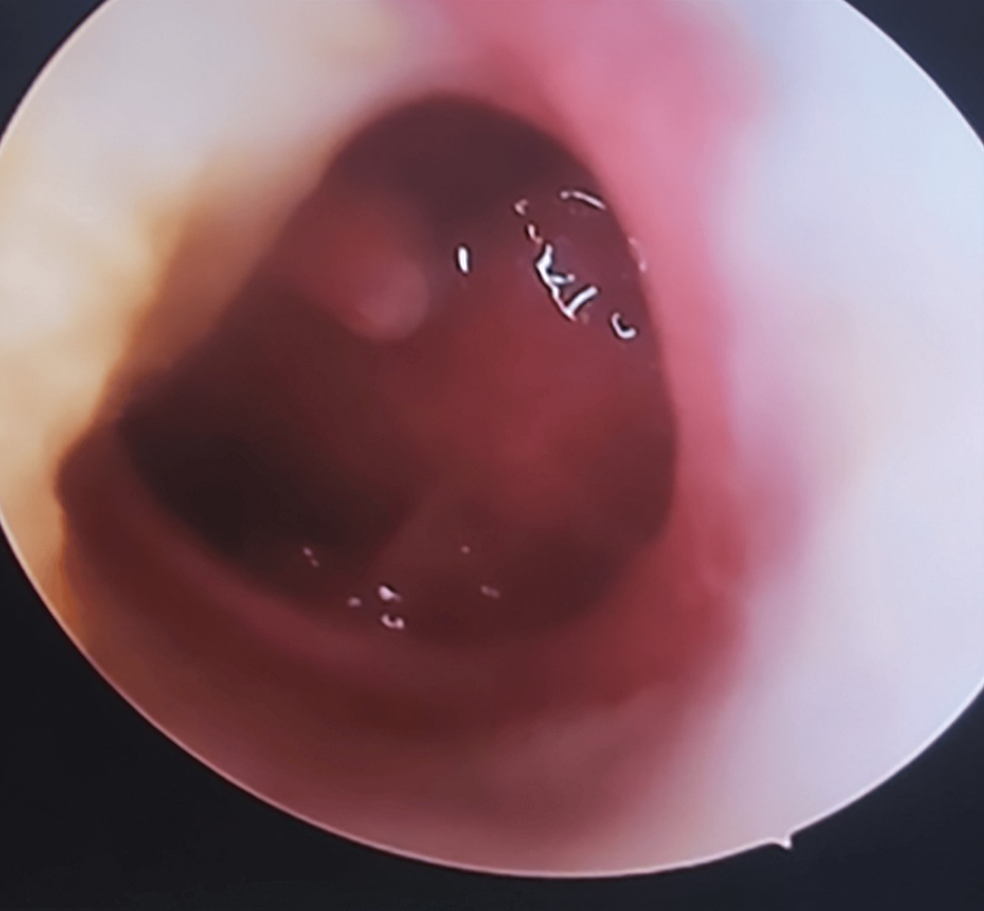
Sub-total tympanic membrane perforation

Out of the 94 cases, the majority, 38 (40.4%) cases, had medium central perforation (Figure 3), followed by sub-total (Figure 4) and large central perforation.

## Discussion

CSOM is an essential contributor to hearing loss, impairments, and poor learning outcomes in children. It can lead to fatal cerebral infections and acute mastoiditis, particularly in countries with limited resources. However, there is little knowledge about the natural development of the disease [9]. *P. aeruginosa* and *S. aureus *are reported as the most often isolated aerobic bacteria [10]. Tropical antibiotics are commonly used in the treatment of the disease. However, the constant administration of antibiotics is rapidly increasing multidrug-resistant organisms, particularly in developing nations [11]. Region-specific local microbial predominance and antibiotic susceptibility studies help decide efficient disease treatment regimens. To evaluate the spectrum of bacterial infection and identify the sensitivity pattern of the isolated organisms, the current study included 94 CSOM cases attending the study centre.

The mean age at presentation was 30.5±16.3 years, with the highest incidence seen in the age group of 11-20 years with 26 (27.7%) cases, followed by the age group of 21-30 years with 24 (25.5%) cases. The findings are in agreement with other similar research [8,12]. In the present study, the male-to-female ratio was 1.35:1. Male preponderance of the disease was also documented in some recent studies [10,13,14]. Of the 94 individuals, 47 had right-sided ear discharge and hearing loss, and 47 had left-sided ear discharge and hearing loss, indicating an incidental matching of 50% involvement of either ear.

The ear discharge was primarily mucoid (n=37, 39.4%) or mucopurulent 28 (n=28, 29.8%), which is similar to the findings of Chowdhury et al. [15]. At the same time, a recent study noted that the pus discharge was mostly purulent and odorous in most patients [10]. With a mean duration of 24.0±14.7 months, ear discharge was prevalent for 13-24 months in one-third of the patients (n= 31, 33.0%), followed by 6-12 months in 22 cases (23.4%). A recent study on chronic otitis media reported a significant association between disease duration and sensorineural hearing loss [16].

Out of the 94 cases, the majority, 38 (40.4%) cases, had medium central perforation, followed by sub-total and large central perforation. The predominance of medium central perforation among CSOM cases agrees with other recent studies [14,17]. The mean air-bone gap among the patients was 26.6±6.7 dB. Pre-operative air-bone gap and perforation size are linked to post-operative hearing enhancement [17,18].

The pattern of bacterial infection revealed a diverse spectrum of isolated organisms among the CSOM cases. Of 94 cases, the gram-positive bacteria constituted 33% (n=31), and gram-negative bacteria constituted 53.2% (n=50) isolates. On the other hand, 11 (11.7%) cases were reported sterile, and yeast cells were isolated in two cases. *S. aureus *was the most common isolated bacterial pathogen (n=30, 31.9%), followed by *P. aeruginosa* (n=26, 27.7%). Other bacterial pathogens isolated included *P. mirabilis*, *K. pneumonia*, *A. baumanni*, *E. coli*, *C. fruendii*, *K. oxytoca* and* A. xyloxidans*. Several other studies have documented a similar spectrum of bacterial profile [3,7,8,19]. Methicillin-resistant* S. aureus* has a high transmission rate in the upper respiratory tract and external auditory canal [19].

On the other hand, *Pseudomonas *is primarily prevalent in tropical regions. It does not typically reside in the upper respiratory tract. Its presence in the middle ear is instead viewed as an additional intruder that enters the middle ear through a defective tympanic membrane and not as an invasion through the eustachian tube [7].

Antimicrobial susceptibility test of the isolated organisms revealed that gram-positive bacteria showed highest sensitivity to cotrimoxazole (67.7%, n=21), followed by clindamycin (48.4%, n=15), ciprofloxacin (45.2%, n=14), azithromycin (41.9%, n=13), tetracycline (38.7%, n=12), gentamycin (35.5%, n=11), linezolid (35.5%, n=11) and mupirocin (32.2%, n=10). On the other hand, amoxicillin-clavulanic acid (51.6%, n=16) and amikacin (58.1%, n=18) showed the highest resistance towards gram-positive bacteria. At the same time, the gram-negative bacteria showed maximum sensitivity to amikacin and ciprofloxacin (78.0%, n=39), respectively. Levofloxacin (32.0%, n=16), amoxicillin-clavulanic acid (28.0%, n=14), cotrimoxazole (26.0%, n=13), gentamycin and piperacillin-tazobactam (24.0%, n=12) also showed sensitivity towards gram-negative bacteria. While clindamycin failed to show any sensitivity, mupirocin (88%, n=44) and azithromycin (60%, n=30) showed the highest resistance towards the gram-negative bacteria strains.

Couzos et al. showed that topical ciprofloxacin is efficacious in community-level treatment [20]. Harshika et al. showed that amikacin, gentamycin and ciprofloxacin were effective against most of the gram-negative bacilli, while gentamycin, doxycycline and chloramphenicol were effective against gram-positive ones [21]. Although quinolones are documented to be effective in the management of otorrhea, recent research emphasizes the use of topical antiseptics along with antibiotics in the management of CSOM cases due to the growing antibiotic resistance of bacterial pathogens [22,23].

Limitations

The present study was undertaken at a single tertiary centre for one year. Multicentric studies with a bigger sample size and longer follow-up periods are required to confirm our findings and examine if conservative treatment of chronic otitis media will stop the disease’s progression and consequences.

## Conclusions

The age at presentation was primarily observed in the second and third decades of life, with the mucoidal ear discharge presenting medium central perforation mainly where the *S. aureus* was the most ordinary isolated gram-positive bacterial pathogen, while *P. aeruginosa* was the most common among the gram-negative bacterial isolates. Gram-positive bacteria showed the highest sensitivity to cotrimoxazole, clindamycin and ciprofloxacin and were resistant towards amoxicillin-clavulanic acid and amikacin and gram-negative bacteria showed maximum sensitivity to amikacin and ciprofloxacin and the highest resistance to mupirocin. The emergence of antibiotic-resistant bacterial strains is growing more widespread nowadays due to the unwise use of antibiotics and the human tendency to discontinue medication before completion of the course, which results in partially resistant microorganisms flourishing. Thus, understanding the pattern of bacterial infection and assessing its antibiotic sensitivity helps decide the proper antibiotic regimen, thereby preventing further difficulties caused by the disease.
